# Effectiveness of Artificial Intelligence Methods in Personalized Aggression Risk Prediction within Inpatient Psychiatric Treatment Settings—A Systematic Review

**DOI:** 10.3390/jpm12091470

**Published:** 2022-09-07

**Authors:** Jing Ling Tay, Ziqiang Li, Kang Sim

**Affiliations:** 1 West Region, Institute of Mental Health, Buangkok Green Medical Park, 10 Buangkok View, Singapore 539747, Singapore; 2 Yong Loo Lin School of Medicine, National University of Singapore, 10 Medical Drive, Singapore 117597, Singapore; 3 Lee Kong Chian School of Medicine, Nanyang Technological University, Clinical Sciences Building, 11 Mandalay Road, Singapore 308232, Singapore

**Keywords:** aggression risk, artificial intelligence, inpatient, prediction, psychiatry, violence risk

## Abstract

Aggression risk assessments are vital to prevent injuries and morbidities amongst patients and staff in psychiatric settings. More recent studies have harnessed artificial intelligence (AI) methods such as machine learning algorithms to determine factors associated with aggression in psychiatric treatment settings. In this review, using Cooper’s five-stage review framework, we aimed to evaluate the: (1) predictive accuracy, and (2) clinical variables associated with AI-based aggression risk prediction amongst psychiatric inpatients. Databases including PubMed, Cochrane, Scopus, PsycINFO, CINAHL were searched for relevant articles until April 2022. The eight included studies were independently evaluated using critical appraisal tools for systematic review developed by Joanna Briggs Institute. Most of the studies (87.5%) examined health records in predicting aggression and reported acceptable to excellent accuracy with specific machine learning algorithms employed (area under curve range 0.75–0.87). No particular machine learning algorithm outperformed the others consistently across studies (area under curve range 0.61–0.87). Relevant factors identified with aggression related to demographic and social profile, past aggression, forensic history, other psychiatric history, psychopathology, challenging behaviors and management domains. The limited extant studies have highlighted a potential role for the use of AI methods to clarify factors associated with aggression in psychiatric inpatient treatment settings.

## 1. Introduction

Patients with psychiatric disorders, including schizophrenia, affective conditions, and substance use disorders, have been associated with a greater risk of aggression [1]. Aggression is defined as a range of hostile behaviors intended to cause harm [2]. Specifically, patients with psychiatric disorders were three to four times more likely than their siblings without psychiatric disorders to be either subjected to aggression or perpetrate aggression [1]. A meta-analysis also found that one in five psychiatric inpatients was assaultive during their hospitalization [3]. Such aggressive episodes can potentially result in physical injuries, prolonged hospitalization and feelings of fear and trauma in victims [4]. Of note, healthcare workers can be victims of such aggression. Ninety-one percent of all healthcare workers, including psychiatrists, psychologists, nurses, social workers and allied health workers, had previously reported experiencing verbal abuse, 45% experienced physical aggression and 23.8% sustained injuries [5]. More than a quarter (26%) of psychiatric nurses suffered serious injuries such as fractures, permanent disabilities or eye injuries during their work dealing with restraints of patients under their care [6]. Consequently, such injuries can aggravate burnout, emotional and psychiatric issues, and affect morale and job satisfaction amongst healthcare workers [7,8]. Being able to predict the occurrence of such aggressive episodes prior to their onset would allow for better preparation to prevent or mitigate the onset and manage the aggressive episode if it occurs.

There are risk assessment tools that have been utilized widely to predict aggression in patients with psychiatric conditions [9]. However, some of these aggression risk assessment tools face limitations in terms of sensitivity and specificity, generalizability to other populations, limited sample size, clinical parameters or data points [9,10,11,12]. Less-valid and reliable aggression risk assessments potentially predispose patients to unfair stigma and discrimination. False positive assessment scores render lower-risk patients to unjustifiable restrictions and higher risk patients to possibly less-warranted medical attention [13].

With the advances in harnessing artificial intelligence (AI) methods to evaluate big data, it is hoped that this may help to address existing limitations and allow for more accurate aggression risk prediction amongst patients seen clinically. Initially used in other areas of medicine, artificial intelligence algorithms, including machine learning methods, have been increasingly evaluated in psychiatry for their feasibility in the: (1) classification of patients from healthy individuals based on composite data in psychotic disorders [14,15] and affective disorders [16]; (2) prediction of depressive disorders [17] and anxiety disorders [18]; and even (3) drug repurposing for potential new treatments in substance use disorders [19]. In aggression risk assessment, predictive analysis can be used to evaluate specific contributory and dynamic factors related to aggression, including personalized data from physiological, movement sensors and electronic health records [20].

Therefore, in view of emerging data on the use of artificial intelligence in psychiatry, especially with regard to feasibility and great potential in aggression risk prediction, we conducted a systematic review and synthesis of the extant literature with two specific aims, namely: (1) to determine the predictive accuracy using artificial intelligence methods for aggression risk prediction amongst psychiatric inpatients, and (2) to elucidate associated clinical factors in predicting aggression risk amongst psychiatric inpatients.

## 2. Methodology

This systematic review was guided by the Joanna Brigg’s Institute (JBI) guidelines for the conduct of systematic review [21]. The methodological rigor in this paper was guided by the five-stage framework [22], namely problem formulation, data collection, evaluation, analysis and interpretation.

### 2.1. Problem Formulation

In this problem formulation stage, we considered the issues related to the use of artificial intelligence methods in predicting aggression within inpatient psychiatric treatment settings. The questions included:

1. What is the predictive accuracy for aggression risk prediction amongst psychiatric inpatients using artificial intelligence methods?
2. What are the associated clinical variables identified in predicting aggression risk amongst psychiatric inpatients?

For the first question, predictive accuracy was determined by measures in relevant studies, including area under curve (AUC), sensitivity, specificity, positive and negative predictive values wherever available.

### 2.2. Data Collection

**Figure 1:**
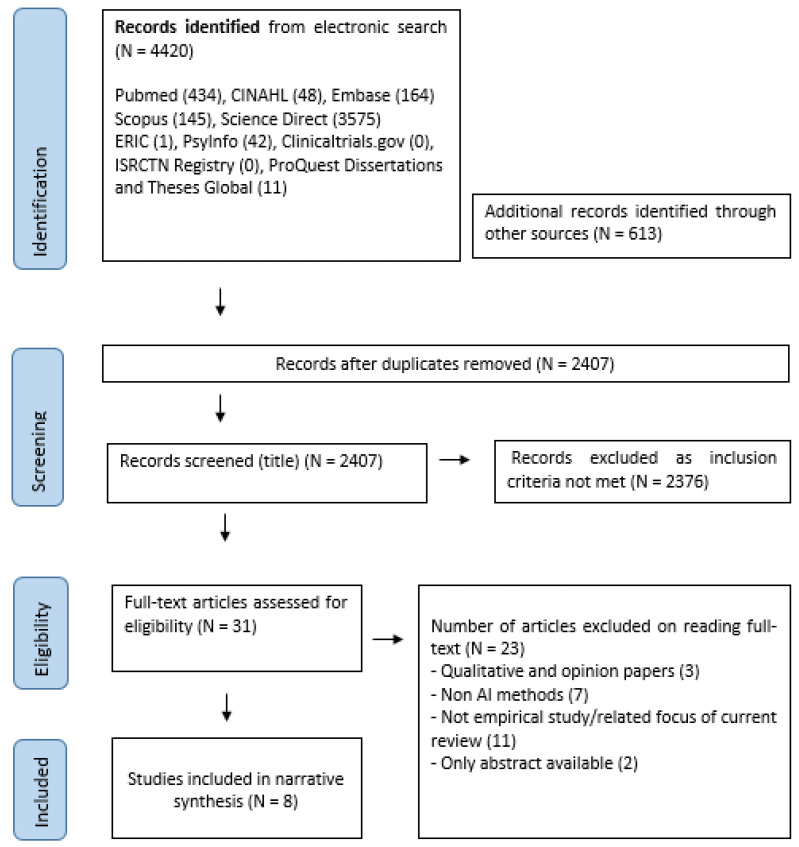
Prisma chart showing search process and final number of papers included in the review.

In this data-collection stage, Pubmed was searched using the key words ‘violen*’ AND ‘inpatien*’ and ‘(artificial intelligence)’. The initial search led to an identification of all keywords. They were ‘(artificial intelligence)’ OR ‘(machine learning)’ OR ‘(natural language processing)’ OR ‘(neural network)’ OR ‘(data science)’ OR ‘(expert* system*)’ OR robot* OR digital* OR technolog* OR device* AND inpatien* AND violen* OR aggressi* OR assault* (Table A1). The team searched for published studies through several electronic databases: PubMed, Cochrane, Scopus, PsycINFO, CINAHL, ERIC, EMBASE, and ScienceDirect. Three databases were searched for unpublished studies: Proquest Dissertation and Theses Global, clinicaltrials.gov and ISRCTN Website. The authors also examined reference lists of review papers, including systematic reviews, meta-analyses, and original research. All empirical studies that were published from inception till the end of April 2022 were included. Please see Figure 1. A total of eight studies were eventually included in this review.

### 2.3. Evaluation (Study Selection and Quality Assessment)

For study selection, inclusion criteria were: (1) target setting was the inpatient psychiatric setting with patient populations across the range of psychiatric diagnoses, (2) use of artificial intelligence methods to predict or manage violence, and (3) only studies in English were included. Qualitative and opinion papers were excluded.

For quality assessment, studies were evaluated for methodological quality by two team members independently. For this stage, we used the Joanna Briggs Institute Critical Appraisal tool for diagnostic test accuracy studies [23]. The inter-rater reliability for the scored items was 75% between the raters. Discrepancies for two papers were settled through thorough discussions within the team and resolved with another independent team member. Overall, the quality scores of the included studies ranged from one to seven (Appendix A).

### 2.4. Data Analysis and Interpretation

Data analysis and interpretation steps were conducted by collating, summarizing, and charting the study findings. For each study, details such as clinical setting, participant or data characteristics, specific measures, if available, and relevant findings were included (see Table 1 for details). In terms of data analysis, statistical pooling was not possible due to the relatively small number of studies and heterogeneity of study designs and patient populations.

## 3. Results

### 3.1. General Features of Included Studies

We evaluated 31 studies for eligibility and excluded 23 studies due to different reasons, leaving eight relevant studies which were included in this review (Figure 1). The methodological qualities of the studies (see Appendix A) were diverse, with quality scores ranging from one to seven.

The included studies were conducted mainly in the West, namely, the United States (*n* = 2), Netherlands (*n* = 2), Switzerland (*n* = 2), Australia (*n* = 1) and Canada (*n* = 1). Participants varied from children, forensic patients to patients with diagnoses including autism spectrum disorder, schizophrenia and mood disorders. Sample sizes ranged from 20 to 358. Five studies described the inclusion of data from electronic health records [24,25,26,27,28], while two studies examined patients’ files [29,30]. The numbers of involved health records ranged from 29,841 to 101,5931 records. Another study captured relevant data for aggression risk prediction using wearable sensors that collected psychophysiological information [31]. The details of the individual studies are summarized in Table 1.

### 3.2. Preparation of Data Points and Process in AI Modelling

Seven studies analyzed data from healthcare records using artificial intelligence models, with five specifically using data from electronic health records [24,25,26,27,28]. These studies described several steps in processing their data for machine learning analysis.

First, texts are extracted for analysis. The texts could be derived from different dictionaries (symptom, sentiment, frequency and diagnosis) [25], guided by existing tools to quantify psychopathological symptoms [29] or proposed by clinicians and data science experts [24,29].

Next, the extracted text underwent further processing [26,27]. The tools used to process the texts included Natural Language Toolkit (NLTK), bag-of-words, Tf-idf (Term frequency–Inverse Document Frequency), word2vec and Paragraph2vec. Alternatively, the extracted texts were recoded as categorical and continuous data, and categorical data were recorded as binary data [29,30].

Variables with massive missing data (>33%) were excluded [29,30], and other missing variables were handled by imputation [29,30]. Conversely, in another study, missing data were dealt with using the creation of ‘missing’ categorical data or via machine learning algorithms such as imputation and data partitioning [24].

Data modelling was conducted with R statistical program [24,29,30] or python [26,28]. Data were split for training and testing. Three studies described this process, where two studies reported using 30% of the data for testing [29,30] while one study used 20% [24]. Four studies highlighted the issue of overfitting [25,27,29,30], which is a machine learning model that fits the training data so well that it impacts model performance on new data [32]. It is the most significant bias that can result from machine learning. Various studies described strategies to minimize overfitting issues such as nested cross-validation [26], nested re-sampling [29,30], five-fold cross-validation strategy, ten-fold stratified cross validation [25], reduction in identified variables [29,30] and using specific algorithms such as elastic net and lasso (least absolute shrinkage and selection operator). Additionally, samples allocated for the testing set were excluded from the training set [25].

Finally, data points were tested using different machine learning models such as logistic regression, decision trees, random forest, gradient boosting, k-nearest neighbor, support vector machines, naïve bayes and evaluated with accuracy markers such as sensitivity, specificity, area under the curve (AUC), positive and negative predictive values.

### 3.3. Accuracy of Artificial Intelligence Methods in Predicting Aggression Risk and Relevant Variables

Our first research question is “What is the predictive accuracy for aggression risk prediction amongst psychiatric inpatients using artificial intelligence methods?”

Most studies reported the Area under the Curve (AUC) value as the accuracy value in the Receiver Operator Characteristics (ROC) curve (Table 2). ROC curve is the graph that plots sensitivity against one minus specificity, which effectively differentiates between true negatives and positives [33]. AUC summarizes the receiver operating characteristic (ROC) curve by providing a measure that differentiates between positive and negative cases. Interpretation of ROC scores were as follows: 0.9 and above (outstanding), 0.80 to <0.90 (excellent), 0.7 to <0.8 (acceptable) and 0.50 to <0.70 (poor discrimination) [34]. The included studies reported acceptable to excellent accuracy with specific employed machine learning algorithms (AUC range between 0.75–0.87). No single machine learning algorithm outperformed the others consistently across the few studies (AUC range between 0.61–0.87).

A study examined the effects of biosensor wearable on wrist [31]. The global model was evaluated by a single classifier containing data from all sessions and participants, whereas person dependent model pertained to personalized evaluation of multiple sessions of a sole participant. It achieved an AUC of 0.71 for the global model and 0.84 for person dependent model. No specific algorithm outperformed consistently amongst studies that utilized AI methods to analyze healthcare records for aggression risk prediction, which included models such as naïve bayes [29], support vector machine [25,27,30], generalized linear model [24], logistic regression [29], random forest, model tree [25] and recurrent neural network [27].

In terms of the factors influencing study findings, one study found that predictive accuracy was greater for earlier rather than later aggression and shorter rather than longer hospitalizations [26]. Results were also influenced by other factors such as the type of text classification and dictionary used. Text embedding was found to perform better than bag-of-words strategies [27]. Document embedding, as compared with other strategies, allowed deep learning models to perform better [27]. The study conducted in Australia found that the sentiment dictionary, which consisted of negative and positive sentiment/ opinion words relevant to emotions, outperformed the symptom dictionary, diagnosis dictionary and frequency dictionary in aggression prediction [25].

### 3.4. Relevant Clinical Variables in Predicting Aggression Risk Amongst Psychiatric Inpatients

Our second research question is “What are the relevant clinical variables identified in predicting aggression risk amongst psychiatric inpatients?” In terms of relevant clinical variables from rating tools and health records identified in the few studies, they can be grouped into factors pertaining to demographic and social profile (e.g., younger and older age, single, childless, fewer years of education, special education, unemployment, problematic work history, financial issues, homelessness, experience of physical neglect); personality (e.g., lower agreeableness); family history (e.g., of suicide); past aggression history (e.g., aggressive threats, witnessed, perpetrated abuse); forensic history (e.g., assault history, poor legal prognosis); other psychiatric history (e.g., depression, suicidal ideation, insomnia, multiple psychiatric admissions); mental status (e.g., uncooperativeness, hostility, irritability, agitation, poor impulse control, psychotic features); rating scales (e.g., high PANSS total scores, positive PANSS score for tension); challenging behaviors (e.g., antisocial behaviors, negative behaviors towards staff and fellow patients, breaking ward rules, complaints about staff); and management domains (e.g., coercive measures needed, time in high security wards, seclusion, haloperidol prescription and higher antipsychotic dose) [24,26,29,30].

## 4. Discussion

Overall, there are few findings from this review. First, whilst the prediction accuracy across tried models and studies had observed an acceptable to excellent range for specific algorithms (AUC range 0.75–0.87), no single machine learning model outperformed the others consistently across the studies (AUC range 0.61–0.87). Second, factors associated with the risk of aggression related to the demographic and social profile, history of aggression, forensic history, other psychiatric histories, mental status and challenging behaviours during admission and management domains.

In terms of accuracy in the prediction of aggression risk based on AUC values, most studies had acceptable to excellent accuracies, but there was no single model that outperformed consistently across the studies. Our findings were comparable (AUC in the acceptable to excellent range) with that of recent studies which employed machine learning models in clinical predictions within inpatient settings related to suicide (AUC 0.77) [35], readmissions (AUC 0.75–0.76), and length of hospital stay (AUC 0.85–0.86) [36]. In our review, only two studies examined the newer supervised deep-learning models [24,27]. The newer supervised machine learning models have incorporated text sequence into their algorithms, and one study found that the deep learning model, especially when coupled with document embedding, achieved slightly better ROC [27] when compared with earlier machine learning algorithms. However, the optimization and balance of data point inclusion and fit of relevant included variables within a specific AI model need further evaluation.

In terms of predictors of aggression, patients with certain demographic and social characteristics were more prone to aggression. The findings in this review were congruent with previous findings which included younger age [37,38,39,40,41,42], older age [43], being unmarried [38,44,45,46], being childless [47], lower education [44], unemployment [44,48,49,50], lower intelligence [38], financial issues [51,52] and homelessness [53]. Of note, an earlier study found an association between homelessness and crimes, but not specifically aggressive crimes [54]. Being subjected to physical neglect was also a predictor of aggression in this review. In contrast, existing literature highlighted other related factors such as physical abuse [55,56], separation from caregivers during growing up years [49], parental abuse and antisocial behaviors towards family members, family illnesses and conflicts [50] as pertinent predictors of aggression.

A common clinical predictor was having prior assaultive history, including aggressive threats, witnessed and perpetrated abuse [24,29,50], which is consistent with extant findings [57,58,59]. Our findings of other aspects in the psychiatric history were also reported in earlier studies, such as depression [40], insomnia [60], suicidal ideations [61,62] and frequent admissions [43]. Like findings from this review, earlier studies had also found that high total PANSS scores predicted aggression [29,50], especially for items such as poor impulse control [63,64], irritability [65], uncooperativeness [66], hostility [41,64] and tension [29,30]. This review found that forensic history and having a poor legal prognosis were predictive of aggression. Likewise, a meta-analysis of 110 studies found that forensic history was the strongest static factor for predicting aggression [64]. In contrast to findings from this review, other studies also observed positive psychotic symptoms [40,50], negative symptoms [50] and poorer insight [40,66] as predictive of aggression.

In terms of the management domain, the usage of haloperidol and high antipsychotic dosage were associated with aggression. The use of haloperidol and high antipsychotic dosage [39] were probably an effect, rather than a cause for aggression [67]. In addition, it was thought that poor compliance with pharmacological and non-pharmacological therapies were correlated with aggression [64], as well as the discontinuation of pharmacological treatment in patients with psychotic disorders such as schizophrenia [50]. In contrast with the current review, other studies also identified additional predictors of aggression, including involuntary admission [68] and off-hour admission [43].

There are several possible inter-relationships between the factors mentioned. For example, homelessness may interact with mental illness, unemployment, need for financial aid and aggression. People with mental illnesses were more likely to be unemployed, aggravating their financial difficulties, which can be associated with homelessness and vice versa [69,70]; homelessness has been independently linked to aggression [71,72]. In addition, the relationship between poorly controlled mental illnesses such as psychotic disorders, level of psychopathology based on PANSS score ratings, and aggression is also plausible. People with poorly controlled psychotic disorders can have more severe psychotic psychopathology and aggression, with higher PANSS total and subdomain scores, and may require involuntary admissions for management of the psychiatric illness and a higher psychotropic dose at the beginning for stabilization [73].

There are several ethical considerations surrounding the use of AI in aggression risk prediction. First is the issue of privacy and surveillance related to principles of respect for persons and non-maleficence. The possibility of such data collection for aggression prediction can potentially translate to blanket surveillance of all patients. Hence, setting certain limits to data access, for example, only on a “need to know and predict basis” for on-duty staff may be useful to protect patients’ privacy [74]. Second, to benefit practical interventions in the clinical settings, evolving clinical context and factors need to be considered when interpreting findings derived from AI platforms and algorithms [74]. Third, any clinical management plan that incorporates data using AI methods to predict and prevent aggression needs to be reviewed over time to ensure that patients are not subjected to unnecessary or unfair seclusion measures.

There are several limitations within this review. First, there were few studies examining the use of AI methods in aggression risk prediction. Second, the heterogeneity of the included studies with the small number of studies to date limited further quantitative analyses, including parcellation of subtypes of aggression. Third, most studies were conducted cross-sectionally and longer-term effects of AI methods in aggression risk prediction were not examined. Fourth, there was also a paucity of data on how AI helps in mitigating and managing aggression in psychiatric inpatient settings over time.

There are several possible future research directions. First, as aggression risk prediction is dynamic; an area where artificial intelligence can be harnessed is its ability to provide iterative and relevant predictions with continual input of current and new data from health records. The dynamic data can potentially shed light on the changing unique clinical profiles of patients related to aggression over time. Second, different machine learning algorithms and models can be combined to better identify longitudinal predictive variables for personalized prevention of aggressive behaviors in inpatient psychiatric treatment settings. Third, incorporating relevant clinical and biological information such as data from clinical assessments, laboratory tests, neuroimaging and neurocognitive assessments can proffer insights into underlying biological factors associated with aggression. It is hoped that the stigma against patients with aggressive tendencies in inpatient settings can be further reduced as we better understand personalized etiological and predictive markers for aggression and reformulate preventive efforts.

## 5. Conclusions

In conclusion, the review revealed that the limited extant studies using machine learning methods had shown its potential to elucidate relevant factors in aggression risk prediction within psychiatric inpatient treatment settings. Further research is needed to investigate the inclusion of additional, longitudinal data points and assess using different machine learning models in order to better understand the inter-relationships between these static and dynamic factors and clinical outcomes over time.

## Figures and Tables

**Table 1:** Details of included studies.

Authors/Year Country/Setting	Patients/ Health Records	Variables Measured	Measure	Main Findings
Goodwin et al., 2019 United States Inpatient setting	Autism spectrum disorder, aged 6–17 years old (*n* = 20)	Wearable wrist biosensor, E4, which measures heart rate, heart rate variability, sweat glands autonomic innervation, and changes in sympathetic nervous system arousal.	Nil	Data were evaluated in cycles of 15 seconds. Aggressive behaviors could be predicted 1 min before they happened, with 3 min of prior biosensor information.
Günther et al., 2020 Switzerland University Hospital of Psychiatry Inpatient Setting	Schizophrenia, with offence history (*n* = 358)	Data obtained from physical records, including psychiatric assessments, treatments and reports from legal documents.	Symptoms were measured with close adoption of Positive and Negative Symptoms Scale, classifying symptoms as present or not.	569 variables narrowed down to 10 predictor variables: aggression threat, actual aggression, prior direct coercive methods, poor impulse control, uncooperativeness, hostility, Haloperidol prescription, higher PANSS scores, higher antipsychotic dosage and unfavorable legal prognosis.
Hofmann et al., 2022 Switzerland University Hospital of Psychiatry Zurich Inpatient Setting	Schizophrenia spectrum disorder (*n* = 352)	Data obtained from physical records including demographic data, social data, childhood and youth history, psychiatric history, criminal history, forensic data, circumstances of current hospitalization and psychopathological symptoms.	Symptoms were measured with the adopted Positive and Negative Symptoms Scale, classifying whether symptoms were absent, partially present or substantially present.	507 probable variables narrowed down to 10 predictor variables: complaints about staff, adverse behaviors towards patients, antisocial behaviors, breaking of ward rules, time at high dependency, higher PANSS score, and adapted PANSS scores for hostility, tension, uncooperativeness and poor impulse control.
Menger et al., 2018 Netherlands University Medical Center Utrecht, Inpatient Setting	Electronic health records (*n* = 1,015,931)	Data from electronic health records written by psychiatrists or nurses in free text format. Psychiatrists’ notes included patient history and treatment. Nurses’ notes included patients’ well-being and activities.	Nil	Classical models like Naïve Bayes and Decision Trees did not achieve equivalent performance with other algorithms, likely due to their simplicity and inability to detect complex patterns.
Menger et al., 2019 Netherlands Inpatient Setting	Electronic health records of patients with psychotic disorders, mood disorders, personality disorders, substance-related disorders Site 1 (*n* = 2209 patients) Site 2 (*n* = 3253 patients)	Data from electronic health records of patients who were admitted. Notes were from 4 weeks prior to patients’ admission up to the initial 24 h of admission. The free text data were converted to numerical form using paragraph2vec algorithm.	Staff Observation Aggression Scale-Revised	Internal validation: Site 1: AUC = 0.80, specificity = 0.94, sensitivity = 0.33 Site 2: AUC = 0.76, specificity = 0.95, sensitivity = 0.34 External validation: Site 1: AUC = 0.72, specificity = 0.93, sensitivity = 0.25 Site 2: AUC = 0.64, specificity = 0.93, sensitivity = 0.13
Suchting et al., 2018 United States Harris County Psychiatric Center, Inpatient setting	Electronic health records (*n* = 29,841)	Data from electronic health records, including demographic data, psychosocial assessment, childhood, education, military and work history, medical and psychiatric history, substance use and treatment, abuse, and financial and living situation.	Affective Disorders Rating Scale	328 probable variables narrowed down to 20 predictor variables: homelessness, forensic assault history, abuse history (witnessed and perpetrated), younger age, aggressive history, lower educational levels, having suicidal ideation upon admission, underwent special education, depressive history, problematic work history, no children, poor sleep, family history of suicide, single, impaired mental state, risk issues, financial difficulties and no prior work history.
Van Le et al., 2018 Australia Wilfred Lopes Centre, forensic inpatient	Electronic Health Records (*n* = 220,000)	Electronic health records of time-sequenced narrative records illustrating observations and comments about each patient. Terms were extracted using different dictionaries: symptom, sentiment and frequency.	Dynamic Appraisal of Situational Aggression (DASA) Historical Clinical Risk Management-20 (HCR-20) Short-Term Assessment of Risk and Treatability (START)	DASA: Support Vector Machine and Logistic Model Tree produced the best models with all three dictionaries. Support Vector Machine had accuracy = 0.77, and LMT had accuracy = 0.75. Best prediction of DASA scores weas from the examination of sentiment language, accuracy 0.56–0.77. HCR-20: Algorithms performed better for symptoms rather than frequency dictionary. START dataset: Root Mean Square Error of 6.29–14.92, and deemed non-reliable.
Wang et al., 2020 Canada Centre for Addiction and Mental Health, Inpatient setting	Schizophrenia spectrum disorder (*n* = 275)	Data from electronic health records, including demographic data, psychiatric history, lifetime alcohol and drug use, suicidal behaviors, personality, experiences of abuse or neglect, family history of mental disorders and suicide.	Modified Overt Aggression Scale Columbia- Suicide Severity Rating Scale Childhood Trauma Questionnaire NEO Five Factor Inventory	Predictors of aggression that were significant included older age (*p* < 0.001), increased hospitalizations (*p* < 0.001), lower agreeableness (*p* = 0.015) and previous history of physical neglect (*p* = 0.042). Predictors of non-aggression included immigration after 18 years old (*p* = 0.033) and family history of mood disorders (*p* = 0.048).

AUC = Area under curve; *n* = number; *p* = value; P = Participant; PANSS = Positive and negative syndrome scales.

**Table 2:** AUCs of machine learning models.

Authors		Machine Learning Models											
	Logistic Regression	Support Vendor Machine	GLM	Random Forest	GBM	K-Nearest Neigbour	Decision Tree	Naive Bayes	J48	Others	NN	RNN	CNN
Goodwin et al., 2019	Global: * 0.71 PD: ** 0.84												
Günther et al., 2020	** 0.85	** 0.84		** 0.86	** 0.84	** 0.80	* 0.79	** 0.85					
Hofmann et al., 2022	** 0.85	** 0.87		** 0.83		** 0.85	** 0.80	** 0.85					
Menger et al., 2018		+BB: * 0.76 +BTI: * 0.76 +WE: * 0.76 +DE: * 0.77					+BB: * 0.73 +BTI: * 0.72 +WE: 0.69 +DE: 0.67	+BB: 0.69 +BTI: * 0.70 +WE: * 0.70 +DE: 0.69			+BB: * 0.73 +BTI: * 0.72 +WE: * 0.75 +DE: * 0.75	+BB: * 0.77 +BTI: * 0.75 +WE: 0.65 +DE: * 0.79	+BB: * 0.73 +BTI: * 0.72 +WE: 0.68 +DE: * 0.76
Menger et al., 2019		Internal validation: * 0.76–** 0.80 External validation: 0.64–* 0.72											
Suchting et al., 2018			* 0.78	* 0.74	* 0.78						* 0.71		
^#^ Van Le et al., 2018	+ST: 0.69 +SY: 0.64 +F: 0.61	+ST: * 0.74 +SY: * 0.70 +F: 0.69		Bagging +ST: 0.70 +SY: 0.68 +F: 0.69			+ST: * 0.75 +SY: * 0.70 +F: * 0.73		+ST: 0.68 +SY: 0.68 +F: 0.59	Jrip +ST: 0.64 +SY: 0.65 +F: 0.63			
Wang et al., 2020	0.64	0.64		**^ 0.63**	0.63 RBF = 0.62					0.64 for Lasso and Elastic net			

Abbreviations: BB = Bag-of-words binary; BTI = Bag-of-words term frequency—inverse document frequency; CNN = Convolutional Neural Network; DE = document embeddings; F = Frequency dictionary; GBM = Gradient boosting machine; GLM = Generalised Linear Model; PD = person dependent; NN = Neural Network; RBF = radial basis function; RNN = Recurrent Neural Network; ST = Sentiment dictionary; SY = Symptom dictionary; WE = word embeddings;. ** excellent AUC value (0.80 to < 0.90), * acceptable AUC value (0.7 to < 0.8) as defined by Hosmer Jr et al., 2013; Bold = best performing algorithm according to AUC; ^ best performing model according to accuracy; # results stated for accuracy.

## Appendix A

**Table A1:** Quality Assessment.

	Bias	1. Consecutive or Random Sampling	2. Case Control Design Avoided	3. Prevent Inappropriate Exclusions	4. Interpret index Results without Knowledge of the Standard Results?	5. Pre-specified Threshold Used	6. Appropriateness of Reference Standard in Classifying Condition?	7. Interpretation of Reference Results without Knowledge of Standard r?	8. Ideal Interval between Index and Reference Results?	9. Same Reference Results for All Patients?	10. Inclusion of All Patients in Analysis	Total Score
Authors												
Goodwin et al., 2019		+	++	+	+	NA	+	+	+	+	++	2
Gunther et al., 2020		+	-	+	+	NA	+	+	+	+	++	1
Hofmann et al., 2022		+	++	++	+	NA	+	+	+	++	++	4
Menger et al., 2018		++	-	++	+	NA	+	+	+	+	++	3
Menger et al., 2019		++	++	++	+	++	++	+	++	++	+	7
Suchting et al., 2018		++	-	++	+	NA	+	+	+	++	++	4
Van Le et al., 2018		++	+	++	+	++	++	+	++	++	++	7
Wang et al., 2020		+	++	++	+	++	+	+	+	++	++	5
NA = not applicable; (-) = high risk; (+) = unclear risk, (++) = low risk												
